# Oral inactivated whole cell vaccine for mucosal immunization: ETVAX case study

**DOI:** 10.3389/fimmu.2023.1125102

**Published:** 2023-03-02

**Authors:** Richard I. Walker, A. Louis Bourgeois

**Affiliations:** Center for Vaccine Innovation and Access, PATH, Washington, DC, United States

**Keywords:** immunity, oral immunization, adjuvant, vaccine, ETEC vaccine

## Abstract

Oral immunization is an effective strategy for inducing protective immunity against mucosal enteric pathogens. Although live-attenuated as well as subunit approaches have been explored for vaccination against enteric pathogens, inactivated whole bacterial cells may also be effective in introducing protective immunity. Successfully accomplishing this goal with inactivated whole bacterial cells will require that a complex antigenic repertoire be presented in controlled immunogenic amounts, in a safe and relatively simple and self-contained delivery format. The benefit from immunization with whole cell vaccines can be further enhanced through genetic engineering to over-express selected antigens and also by the use of mucosal adjuvants to direct a more robust immunologic response. These steps are being taken for the development of ETVAX, the most clinically advanced vaccine candidate against the major enteric pathogen, enterotoxigenic *Escherichia coli* (ETEC) with significant positive impact.

## Introduction

Enterotoxigenic *Escherichia coli* (ETEC) remains among the most common bacterial causes of diarrhea-associated morbidity and mortality and is often the first bacterial illness that children experience in endemic areas (1, 2). It is an enterotoxic non-invasive disease in which bacteria must first colonize the small intestine where they employ plasmid-encoded fimbrial colonization factors (CFs) or coli surface antigens (CS) to bind enterocytes in the upper small intestine (1, 2). Here they produce heat-stable (ST) and/or heat-labile (LT) enterotoxins, and their close association with enterocytes *via* their CF/CS antigens promotes transfer of ETEC enterotoxins that stimulate the release of fluid from the intestinal epithelium, resulting in watery diarrheal illness (1, 2). Even asymptomatic intestinal colonization with ETEC strains can lead to local and systemic inflammation that further highlights ETEC as a contributor to the pathogenic pathway leading to stunting EDD and poor cognitive development (1, 2).

There are no licensed vaccines for ETEC. However, field studies and human challenge studies indicate that protective immunity to ETEC develops after natural or experimental infection (1, 2). In addition, in ETEC-endemic areas, age-specific attack rates for symptomatic ETEC infection decline after three years of age (1, 2). ETEC vaccine development efforts have focused on the induction of anti-toxin and anti-colonization immunity, including using whole cell inactivated or live attenuated, purified antigens, subunit and peptide approaches (1, 2). Specifically, studies in animals and human subjects indicate that LT and CF/CS antigens contribute to protection against ETEC and have potential for use in a vaccine. The currently most advanced vaccine candidate for ETEC, ETVAX, employs these antigens (1, 2).

## Optimization of antigenic content for an inactivated whole cell ETEC vaccine candidate

The development of ETVAX may provide a valuable roadmap for development of other improved inactivated whole cell enteric vaccines (1, 2), and I. Khalil, personal communication. A first-generation precursor of the current ETVAX candidate was a whole cell vaccine consisting of formalin-inactivated ETEC bacteria expressing CFA/I and CS1 to CS5, combined with cholera toxin B-subunit (CTB), which is highly homologous to the B-subunit (LTB) of LT enterotoxin produced by approximately 66% of ETEC strains (3). This 2-dose vaccine candidate was found to be immunogenic in children and adults in endemic areas. Furthermore, it protected adult travelers against moderate/severe diarrhea (4–6). In these studies, an anti-CTB serum IgA titer > 360 when trial participants arrived in Guatemala or Mexico was found to be associated with a reduced risk of developing moderate to severe ETEC traveler’s diarrhea (TD) (**Table 1**), suggesting that level of anti-toxoid IgA antibody may be a marker for effective vaccination in travelers. In pediatric studies, a full dose of the vaccine caused vomiting in 6–17 months old Bangladeshi children; but a quarter dose was found to be safe (7). The vaccine did not confer protection in 6–24 months old Egyptian children, but both vaccinated and unvaccinated children experienced mainly mild disease during the study period and the impact on moderate/severe diarrhea could not be evaluated (8). These data were reviewed by a WHO panel, which recommended overexpression of antigens on the cells comprising the vaccine and inclusion of an adjuvant in the formulation (9) to help improve vaccine immunogenicity in the target age-group.

**Table 1:** Anti-CTB serum IgA serum IgA titer^1^ in U.S. travelers upon arrival in Guatemala or Mexico and the risk of developing moderate to severe ETEC traveler’s diarrhea.

Anti-CTB IgA Titer at Arrival	Number of Travelers	Mod-Sev TD VPO	No of Mild TD VPO	Relative Risk^2,3^
≤40	279	10	269	Reference
41-120	31	2	29	1.80 (0.41, 7.85)
121-360	73	2	71	0.76 (0.17, 3.41)
361-1080	81	0	81	0.16 (0.01, 2.75)
>1080	185	1	184	0.15 (0.02, 1.17)

^1^Range of anti-CTB titers in arrival serum samples collected from US adults traveling to Guatemala and Mexico regardless of vaccination status. Subjects with reciprocal anti-CTB titer ≤40 were negative.

^2^Cochran-Armitage test tor trend (p-value=0.01) using dichotomous cut-point at 360, RR=0.10 (95%CI:0.01-0.78) p=0.014.

^3^Mild vaccine preventable ETEC Traveler’s diarrhea (Mild ETEC VPO TD) = ETEC recovered from subjects as a sole pathogen during TD episode, sharing an antigen(s) with the ETEC vaccine (LT, CFA/I, CS1, Cs2, CS3, CS4, and CS5; the vaccine did not contain immunogenic quantities of CS6); 3 unformed or liquid stools in a 24 hr period accompanied by abdominal pain/cramps, nausea and or vomiting of any intensity; Moderate to severe VPO TD = ETEC recovered from subjects as a sole pathogen during TD episode; ≥ 3 unformed or liquid stools in a 24 hr. period and moderate to severe GI symptoms or illness causing a change in normal daily activity.

## Swedish trial of second-generation vaccine

ETVAX is now undergoing clinical trials as a second generation multivalent oral ETEC vaccine-containing inactivated *E. coli* strains over-expressing colonization factor antigens CFA/I, CS3, CS5 and CS6 (the latter was not included in the first-generation vaccine) at significantly higher levels than in naturally occurring cells (10). In addition, it was also noted that formalin inactivation of the CS6 expressing ETEC was detrimental to CS6 immunogenicity, and this was overcome in the second-generation formulation by phenol inactivation of the CS6 strain (10). The current generation vaccine is administered together with the more LT-like toxoid LCTBA (11–13). To further enhance the immunogenicity of the vaccine, it was combined with the double mutant labile toxin of ETEC (dmLT) adjuvant (10, 14, 15). When tested in Swedish adults (ISRCTN91363076), ETVAX with or without dmLT was found to be safe and to induce significant fecal SIgA responses as well as IgA antibody-secreting cell (ASC) responses against all CFs and LTB (12). Addition of 10 ug dmLT to the vaccine significantly enhanced ALS responses only to the CS6 component (12). ETVAX also induced long-lasting immunological memory in Swedish adults (16; ISRCTN27096290). Subsequent results demonstrated that ETVAX induced IgA antibody responses that cross-react with CFs belonging to the same CF families, possibly expanding the coverage of the vaccine (17). These successful results have led to clinical evaluation of ETVAX in a large phase 1/2 trial in adults and lower age groups (5 years to 6 months) in Bangladesh.

## Use of an adjuvant to maximize immune responses in vaccine target age-group

### Bangladesh trials

Adults: The safety of ETVAX alone or together with dmLT adjuvant was evaluated in Bangladeshi adults to provide the basis for studies in Bangladeshi children and infants [(3); NCT02531802]. The Bangladeshi adults received two oral doses of ETVAX with or with-out dmLT adjuvant or placebo in the initial part of a randomized, double-blind, placebo-controlled, age-descending, dose-escalation trial. This was the first clinical trial of the second generation ETVAX vaccine conducted in an ETEC endemic country. Two full doses of ETVAX, both when administered alone and with 10 ug dmLT adjuvant, were safe and well tolerated in the healthy Bangladeshi adults tested, confirming previous safety data from studies in Swedish adults (11, 12, 16). This vaccine was highly immunogenic in Bangladeshi adults, inducing intestine-derived ASC responses in all, and plasma antibody responses in a majority, of the vaccine recipients to all primary vaccine antigens (four CFs and LTB). Addition of dmLT adjuvant to the vaccine had no apparent effect on the ALS responses in the adults in this study. In contrast, in adult Swedes, 10 ug of dmLT significantly enhanced ASC responses to CS6, which is the CF present in the lowest amount in the vaccine (12).

Both the Swedish and Bangladeshi trials demonstrated that the oral ETVAX vaccine is safe in adults and induces strong mucosal as well as systemic immune responses against key vaccine antigens. These findings have provided a base for further evaluation of this vaccine in descending age groups in children and infants.

Children and infants: Healthy children in one of three age groups (24–59 months, 12–23 months, and 6–11 months) were randomly assigned with block randomization to receive either ETVAX, with or without dmLT, or placebo (18; NCT02531802). ETVAX (half [5.5 × 10^10^ cells, 500 ug LCTBA], quarter [2.5 × 10^10^ cells, 250 ug LCTBA], or eighth [1.25 × 10^10^ cells, 125 ug LCTBA] adult dose), with or without dmLT adjuvant (2.5 μg, 5.0 μg, or 10.0 μg), or placebo were administered orally in two doses 2 weeks apart. The primary endpoint was safety and tolerability, assessed in all children who received at least one dose of vaccine. Antibody responses to vaccine antigens, defined as at least a two-times increase in antibody levels between baseline and post-immunization, were assessed as secondary endpoints.

No solicited adverse events occurred that were greater than moderate in severity, and most were mild. The most common solicited event was vomiting which appeared related to dose and age. The addition of dmLT did not modify the safety profile. Mucosal IgA antibody responses in lymphocyte secretions were detected against all primary vaccine antigens (CFA/I, CS3, CS5, CS6, and the LCTBA toxoid) in most participants in the two older age groups, whereas such responses to four of the five antigens were less frequent and of lower magnitude in infants aged 6–11 months than in older children.

Mucosal antibody response frequencies were consistently higher in the adjuvanted vaccine group when ≥ 3, 4 or 5 antigens were considered (**Table 2**). Seventy-eight (56%) of 139 infants aged 6–11 months who were vaccinated developed mucosal responses against at least three of the vaccine antigens versus 14 (29%) of 49 of the infants given placebo. Addition of the adjuvant dmLT enhanced the magnitude, breadth, and kinetics (based on number of responders after the first dose of vaccine) of immune responses in infants (19). A similar positive dmLT effect was observed on the mucosal antibody response in the 12-23 month age group in this study when the antigenic breath of the responses was considered. In more in-depth immunological studies, the inclusion of dmLT in the vaccine formulation improved expression of B cell memory markers and T cell responses to key ETEC antigens as well (19–21). Subsequent findings showed that ETVAX induced mucosal and systemic immune responses against O78 LPS (present in three of the CS expressing strains in the vaccine) in all age groups and that dmLT improved intestinal immune responses among infants (19, 22). These observations may have implications for more successful use of dmLT with other oral vaccines, like cholera (OCV), which have experienced difficulty in inducing protective O antigen antibody responses in children and infants under 5 years of age (23). Also, the enhancement of protein colonization factors and 078 LPS responses in infants and young children suggests in the case of OCV that including dmLT in the formulation may make single dose vaccine strategies more protective, thereby extending available stockpiles of this vaccine (24).

**Table 2:** Cumulative frequencies of 6-11 months old children given a 1/4 dose of vaccine alone or with 2.5 µg of dmLT with ≥2 fold fecal and/or ALS IgA responders against different numbers of key ETVAX vaccine antigens.

	Placebo (n=30)	¼ Dose ETVAX (n=30)	¼ Dose ETVAX + 2.5ug dmLT (n=28)
**5 Antigens, n(%)**	0(0%)	7(23%)	12(43%)
**>4 Antigens, n(%)**	3(15%)	10(33%)	15(54%)
**>3 Antigens, n(%)**	3(15%)	10(33%)	20(71%)

Responder frequencies were significantly higher by Fisher’s exact test in the ETVAX + dmLT versus placebo groups for 5 antigens (p=0.0005), ≥4 antigens (p=0.0078), and ≥3 antigens (p=0.001). Responder frequencies were also significantly different between the vaccine alone vs placebo groups for 5 antigens (p=0.03), but not for ≥4 or ≥3 antigens.

The Swedish and Bangladeshi studies showed that ETVAX could induce strong intestine-derived and/or fecal immune responses in a majority of vaccinated adults and in different age groups, including infants, in Bangladesh (21). This trial assessed for the first time the safety and immunogenicity of ETVAX in young children and infants in a low and middle income country (LMIC) and was the first analysis of dmLT adjuvant with a vaccine in this population in a LMIC. Immune responses were improved in infants aged 6–11 months by administering dmLT with the vaccine. Importantly, this study showed that occasional mild to moderate vomiting can be reduced without loss of immunogenicity by reducing the dose of vaccine, even when dmLT was included in the formulation.

### Zambian trials

A double-blind, placebo-controlled, age-descending, dose-finding trial was undertaken in 40 adults, 60 children aged 10-23 months and 146 aged 6-9 months old (25; PACTR201905764389804). Adults received a single full dose of ETVAX and children received 3 doses of either 1/4 or 1/8 of the vaccine. All vaccine doses also included 2.5 ug of dmLT. There were no differences in the frequency of solicited or unsolicited adverse events between vaccine recipients receiving 1/4 and 1/8 dose and no increase in frequency or severity of systemic reactogenicity with increasing number of vaccinations or decreasing age. The vaccine induced plasma IgA and IgG responses against LTB in 100% of the adults and 80-90% of the children. In children aged 6-9 months, IgA response rates against ≥ 4 vaccine antigens after 3 doses determined as ≥ 2-fold and ≥ 4-fold rises were significantly higher for 1/4 dose compared to placebo (65.1% vs 27.6%, p=0.004 and 32.6% vs 6.9%, p=0.01, respectively).

The Zambian studies further demonstrated that ETVAX was safe, tolerable, and immunogenic in adults and children. Using 3 doses, the maximum expected number of doses to be used in children in LMICs, the 1/4 dose was again shown to be safe and induced significant IgA responses in children.

Evaluation of pre-and post-vaccination plasma samples from 20 children aged 10-23 months was accomplished using a proteome microarray (26). Post-vaccination, reactivity to CFA/1, CS3, CS6, and LTB was stronger than baseline among the vaccinated compared to the placebo group. The CS5 protein was not included in the microarray. Three other purified non-vaccine ETEC proteins; CS4, CS14, and PCF071 had significantly higher post-vaccination responses. These data suggest that ETVAX induces cross-reactive IgG antibody responses to non-vaccine CFs CS4, CS14, and PCF071 from the class 5 fimbriae. This activity could provide some broad IgG antibody coverage beyond the core vaccine antigens (CFA/I, CS3,CS5, CS6 and LTB) themselves.

Field evidence that effective immunization with ETVAX can be protective in man.

### Benin trial

A randomized, double-blinded, placebo-controlled phase 2b trial of ETVAX supplemented with 10 ug of dmLT was conducted among 729 Finnish volunteers (18-65 years old) randomized to receive two doses of either ETVAX or placebo two weeks apart (27; EudraCT2016-002690-35; NCT03729219). Although the data for this trial are being prepared for initial publication elsewhere, it can be said that the safety, immunogenicity and efficacy data are encouraging for moving on to Phase 3 planning for travelers and further studies among infants and young children in low to low-middle income countries (LMICs) that will lead to Phase 3 testing for both indications. The Benin Phase 2B study was a critical step in the development of the ETVAX vaccine since it was a very stringent test of its impact on ETEC TD since the burden of co-pathogens in the field proved exceptionally high and ETEC was found in 75% of all severe diarrhea cases. These data for protection are consistent with those reported for the first generation inactivated ETEC vaccine (4–6).

### Gambian trials

As described above, ETVAX has been shown to be safe and induce strong intestinal-mucosal IgA antibody responses to CF antigens and LTB when tested among adult Swedish volunteers, healthy Bangladeshi adults and children, and in healthy Zambian children. An ongoing trial in The Gambia is a randomized, double-blind, placebo controlled (1:1) trial to measure the safety, immunogenicity, and protective efficacy of three ETVAX doses given as 1/4 of an adult dose plus 2.5 ug of dmLT. Vaccine is given on days 1, 15, and 90 to healthy Gambian children aged 6 to 18 months (28; PACTR20201081021852). As of 17 November 2022, 4936 children have been enrolled and received at least one vaccine dose and 88% of these children received all three doses. Although safety data is blinded to group assignment, the investigational product appears safe. Active surveillance using home visits for solicited adverse events (i.e., diarrhea, vomiting, fever, acute allergic reactions) within seven days of any vaccine or placebo dose in a cohort of 350 children detected 89 events, only two events (1 vomiting, 1 fever) were severe and considered product related. Among all other children (n = 4115), there were 348 adverse events during dosing; three were severe (1 pneumonia, 1 measles, 1 bronchiolitis) and none were product related. For 32 serious adverse events, none were found product related. To 31 October 2022, 360 moderate to severe diarrhea cases have been detected and 94 (26%) were ETEC positive by multiplex PCR. Stool specimens are under testing for ETEC phenotypes and co-pathogens.

## Practical oral delivery of whole cell vaccines

To date the only licensed inactivated whole cell vaccines against enteric diseases are against cholera, and most depend upon delivery of acid-resistant polysaccharide antigens. The presentation for these vaccines not requiring a buffer involves a cell suspension in a plastic tube that can be dispensed directly into the recipient’s mouth. A more complex presentation may be necessary to exploit the potential of whole cell vaccines containing adjuvants and conserved protein antigens. This need has been studied extensively with ETVAX so that the number of vaccine components and their presentation for administration can be simplified (N. Carlin, personal communication). The presentations now being considered for traveler and LMIC pediatric use of ETVAX will consist of a spray dried sachet (dmLT [10 ug] + antacid buffer + LCTBA) and a liquid bottle of 10 ml of bacterial cells For adults the sachet may be reconstituted in the bacterial suspension of cells in a glass containing 150 ml of water prior to consumption. For children in LMICs, the sachet contents are anticipated to be mixed directly with the bacterial suspension for administration. The two-component, self-contained formulation is a significant advance. The need for no extraneous water overcomes a delivery issue that has been problematic for widespread use of some oral inactivated whole cell vaccines (i.e.Dukorall). At this point, ETVAX has a practical formulation designed to minimize user error. Future work may be able to further reduce the footprint of this vaccine for refrigeration.

## Potential for a multi-pathogen vaccine

Although ETVAX promises to be an effective vaccine with broad protection against ETEC, the value proposition for this vaccine could be greatly increased by using it as a platform to immunize against other pathogens (2); I. Khalil, personal communication). For example, recombinant protective antigens against other mucosal pathogens could be cloned into one or more of the strains now comprising ETVAX. Alternatively, other whole cell vaccines alone or themselves expressing heterologous antigens could be co-formulated with ETVAX. These could include the cross-protective inactivated *Shigella* whole cell vaccine (29) or the Hikojima (30) or Euvichol (31) cholera vaccines. An advantage of a practical means of oral vaccine delivery compared to parenteral injections is that the other whole cell vaccines would not need to be mixed before administration to be acceptable. Instead, after administering the ETVAX with buffer and adjuvant, a second simpler plastic vial containing just the additional whole cell antigens in PBS could be used for further oral immunization, possibly benefitting from the buffer and adjuvant in ETVAX.

## Concluding remarks

The data obtained through the development of ETVAX clearly show that an effective and practical inactivated whole cell vaccine can be adapted for general use. Not only are whole cell vaccines immunogenic, but genetic engineering, as shown with ETVAX, can improve their immunogenicity in both infants and travelers. Further, the utilization of dmLT mucosal adjuvant may help overcome the weaker responses to oral vaccines often encountered with oral immunization of young children living in LMICs. The safety of Gram negative whole cell vaccines in young children can be improved with dose reduction without significant losses of immunogenicity. Finally, much progress has been made towards practical oral vaccine presentations. It is hoped that these experiences to date with ETVAX will facilitate not only its development but also the development of other much-needed whole cell-based vaccines such as for cholera and *Shigella* for oral administration alone or in combinations.

## Data availability statement

The original contributions presented in the study are included in the article/supplementary material, further inquiries can be directed to the corresponding author/s.

## Ethics statement

The studies involving human participants given ETVAX referenced in this perspective were reviewed and approved by the appropriate institutional ethics committees:

- OEV-121 (Sweden): The Ethical Review Board in the Gothenburg Region, the Western Institutional Review Board, USA and the Swedish Medical Product Agency (Eudract no. 2011-003228-11).
- OEV-122 (Bangladesh): The Declaration of Helsinki and approved by the Research Review and Ethical Review Committees of the International Centre for Diarrhoeal Disease Research, Bangladesh; the Western Institutional Review Board, USA; and the US Food and Drug Administration (clinicaltrials.gov NCT02531802).
- OEV-123 (Benin): Coordinating Ethics Committee of Hospital District of Helsinki and Uusimaa (Finland) (clinicaltrials.gov NCT03729219).
- OEV-124 (Zambia): Ethics committee UNZABREC: University of Zambia Biomedical Research Ethics Committee (PACTR201905764389804).
- OEV-125 (Sweden): Etikprövningsmyndigheten (Swedish Ethical Review Authority) (Sweden) and Human Research Protection Office USAMRDC Office of Research Protections (ORP) (USA) (clinicaltrials.gov NCT05178134).
- OEV-128 (The Gambia): The Gambia Government/MRC Join Ethics Committee, Observational/Interventions Research Ethics Committee., London School of Hygiene and Tropical Medicine, Medical Control Agency (MCA) (PACTR202010819218562). Written informed consent to participate in these studies was provided by the patient/participants’ OR patient/participants legal guardian/next of kin.

## Author contributions

The first draft of the manuscript was written by RW and reviewed and further edited and added to by AB. All authors contributed to the article and approved the submitted version.
